# Navigating biosafety concerns within COVID-19 do-it-yourself (DIY) science: an ethnographic and interview study

**DOI:** 10.1057/s41292-023-00301-2

**Published:** 2023-03-28

**Authors:** Anna Wexler, Rebekah Choi, Alex Pearlman, Lisa M. Rasmussen

**Affiliations:** 1 Department of Medical Ethics and Health Policy, University of Pennsylvania, 423 Guardian Drive, Philadelphia, PA 19104 USA; 2 Department of Philosophy, University of North Carolina at Charlotte, Charlotte, USA

**Keywords:** DIY science, DIY biology, Biomedical citizen science, Bioethics, COVID-19

## Abstract

Non-establishment or do-it-yourself (DIY) science involves individuals who may not have formal training conducting experiments outside of institutional settings. While prior scholarship has examined the motivations and values of those involved in the subset of DIY science known as “DIY biology,” little research has addressed how these individuals navigate ethical issues in practice. The present study therefore aimed to understand how DIY biologists identify, approach, and resolve one particular ethical issue—biosafety—in their work. We conducted a digital ethnography of Just One Giant Lab (JOGL), the primary hub for DIY biology during the COVID-19 pandemic, and subsequently conducted interviews with individuals involved with JOGL. We found that JOGL was the first global DIY biology initiative to create a Biosafety Advisory Board and develop formal biosafety guidelines that applied to different groups in multiple locations. There was disagreement, however, regarding whether the Board should have an advisory role or provide mandatory oversight. We found that JOGL practiced ethical gatekeeping of projects that fell outside the limits defined by the Board. Our findings show that the DIY biology community recognized biosafety issues and tried to build infrastructure to facilitate the safe conduct of research.

**Supplementary Information:**

The online version contains supplementary material available at 10.1057/s41292-023-00301-2.

## Introduction

Biomedical research is most commonly conducted in institutional settings by researchers with formal training. By contrast, what has been termed “non-establishment research” (Rasmussen et al. 2020) involves individuals who may not have scientific training conducting experiments outside of traditional settings (Landrain et al. 2013; Meyer 2013). This research has been alternately referred to as “do-it-yourself science” (Ferretti 2019), “biomedical citizen science” (Wiggins and Wilbanks 2019; Guerrini, Wexler, et al. 2019; Trejo et al. 2020), “biohacking” (Delfanti 2013; Wexler 2017), “participant-led research” (Kempner and Bailey 2019; Vayena et al. 2016; Grant et al. 2019), or “community biology” (Walker et al. 2020). It includes practices as varied as hacking diabetes devices (Lee et al. 2016; Omer 2016), self-administering unapproved medical treatments (Ekekezie et al. 2020; Wexler 2016), developing treatments for disease (Kempner and Bailey 2019), and experimenting with gene-editing techniques (Pauwels and Denton 2018).

This paper focuses on the subset of non-establishment research that has been termed “DIY Biology” or “DIYbio” (Meyer 2013; Landrain et al. 2013; Sundaram 2021), which encompasses a loose-knit group of individuals undertaking a wide range of activities related to biology, which they conduct at home or in local biology labs (Erikainen 2022; Delfanti 2013). Many of those involved with DIY biology refer to themselves as a “community” or as a grassroots movement, and have built relationships with one other, through both in-person interactions (e.g., at community labs and at national and international meetings) as well as via extensive online communication (Wexler 2017; Meyer and Vergnaud 2020; Seyfried et al. 2014). As there is no consensus regarding terminology even among participants (Trejo et al. 2020), here we utilize the term DIY Biology or DIYbio when discussing the abovementioned group, and DIY science when referring to citizen science writ large.

Because DIY science is not typically federally funded or conducted at federally sponsored institutions, there are no systematic layers of regulatory oversight to ensure that it has met particular ethical standards (Rothstein et al. 2015; Guerrini et al. 2018; Rasmussen 2021). In the absence of regulations, many have argued that the ethical conduct of DIY science poses unique concerns for DIY communities, policymakers, and conventional research ethics gatekeepers (Edwards 2017; Grant et al. 2019; Vayena and Tasioulas 2013b; Rasmussen 2017; Fiske et al. 2018; Wiggins and Wilbanks 2019; Resnik 2019). Some have proposed that a new “social contract” is needed to enable this kind of research and minimize its pitfalls (Vayena et al. 2016) and have developed frameworks illustrating how ethical issues may differ depending on the degree of involvement of lay individuals (Vayena and Tasioulas 2013a). Other work has assessed DIY biologists’ attitudes toward ethics, finding that they prioritize issues such as failure to return results and power imbalance (Guerrini et al. 2021), and that they are most open to a form of ethical oversight that is voluntary and community-driven (Trejo et al. 2021).

One of the most frequently expressed ethical concerns regarding DIY biology is that of biosafety. Concerns have been raised that individuals may cause physical harm to themselves (i.e., by conducting unsafe experiments) or to the public, due to the risk of pathogens escaping the laboratory, either accidentally or deliberately (Kuiken 2016, 2020; Kolodziejczyk 2017; Lim 2021; Sundaram 2021). Those involved with DIY biology have responded to such concerns by pointing to the long history of efforts toward biosafety within the movement, which has included locally established biosafety codes, the “Ask a Biosafety Expert” feature on DIYbio.org, and a biosafety training camp that featured involvement from a leading international biosafety organization (Sundaram 2021; Grushkin et al. 2013; Kuiken 2016; Grushkin 2018). Still, efforts to quell biosafety concerns have done little to quash them. Recently, the COVID-19 pandemic brought renewed attention to issues of biosafety within DIY biology, with the media and scholars expressing concerns about the dangers of self-experimentation with DIY vaccines (Caplan and Bateman-House 2020; Shah and Jamrozik 2020; Guerrini, Sherkow, et al. 2020; Murphy 2020).

To date, despite the longstanding public interest in biosafety within DIY science, there has been little empirical research on how citizen scientists navigate biosafety issues in practice. While prior sociological scholarship has examined the motivations, values, and politics of DIY biology (Meyer 2013; Barba 2014; Delgado and Callén 2017; McGowan et al. 2017; Roosth 2017; Grant et al. 2019; Guerrini, Trejo, et al. 2020) and other work has cataloged the history of biosafety efforts within DIY biology (Grushkin et al. 2013; Kuiken 2016; Sundaram 2021; Lim 2021), this work has not examined how individuals anticipate and respond to biosafety challenges in their own scientific investigations.

The present study aimed to fill this gap by empirically assessing how individuals involved in DIY biology navigated challenges related to biosafety. The COVID-19 pandemic offered a unique setting to examine this question, as it spurred efforts from DIY biologists to coordinate—virtually and globally—on efforts to develop COVID-19 diagnostics and preventatives. Because those involved in DIYbio were forced to communicate primarily via online outlets, both out of geographic necessity and due to quarantine restrictions, there was rich ground for digital ethnography. In addition, the international nature of DIY efforts during the pandemic meant that participants did not necessarily share a common ethical framework, thereby offering an opportunity to study issues as they arose in a global setting that was not dependent on shared ethical values.

The present study employed a two-phased approach. In the first phase, we conducted a digital ethnography of Just One Giant Lab (JOGL), the online platform that became the de facto hub for DIYbio during the COVID-19 pandemic. In the second phase, we sought to gain a deeper understanding of our data by conducting follow-up interviews with those involved in biosafety-related issues on the JOGL platform. Our aim was to advance knowledge of how those involved in DIY biology identify, approach, and resolve biosafety-related considerations in their work.

## Methods

### Just One Giant Lab (JOGL)

Just One Giant Lab (JOGL) is an online platform that was launched in 2019 by three longstanding members of the DIY biology community to foster open science collaboration across borders (JOGL 2022). In February 2020, the platform introduced the “OpenCovid19 Initiative,” a dedicated area of the site where individuals could network with others to address COVID-19 issues across five broad areas: diagnostics, validation, treatment, prevention, and data (JOGL 2020a). Individuals could freely create personal profiles as well as “project pages” that described specific endeavors, such as efforts to develop a one-hour diagnostic test, face shields made from recycled plastic, and an app that would use machine learning to compare a normal cough to that of someone infected with COVID (Jorgenson 2020; Lowe 2021; Bektas 2020; Morales 2020). Given the lack of a messaging feature on the JOGL platform, project participants began turning to Slack, a popular workplace communication tool, to converse with one another. Though Slack was initially designed for the quick exchange of messages, both in public and private “channels,” as well as through direct messages, JOGL participants began utilizing Slack for their growing project needs. Activity on the JOGL Slack channel peaked in late March 2020, when there were approximately 600 active members, nearly two thousand registered users, and several dozen channels, which were dedicated both to individual projects and general topics (e.g., events, announcements, onboarding, community, “looking for resources,” and “looking for skills”). Hundreds of new members flooded the JOGL Slack every few days, and by April 2020, Slack had become the primary communication method for all JOGL participants.

### Phase 1: digital ethnography

Due to the large volume of conversations across JOGL Slack channels, we employed several methods to narrow our focus to biosafety-related content. First, we met with JOGL leadership and described our project at a weekly public meeting; through this and by browsing JOGL Slack channels, we identified entire channels of interest (such as one dedicated entirely to biosafety). Second, keywords related to biosafety were developed iteratively by all four authors in a process that involved generating terms, revising them during group discussion, testing them for relevance on the JOGL Slack channels, and revising again until all authors concurred with the final keyword list (see Appendix 1). Next, all conversations occurring on public Slack channels between March 1 and August 31, 2020 were searched using these keywords, and conversational snippets occurring before and after the use of this keyword were captured. To make JOGL participants aware of our research, we announced our project at a weekly JOGL Zoom call and pinned a post in the public JOGL Slack announcement channel during data collection (August-December 2020), offering users the opportunity to opt out of having their textual data collected.

Search results for each keyword were compiled into individual documents, which were then reviewed, annotated, and discussed jointly by all authors. For both methods (whole channel review and conversational review based on search terms), we sought out and reviewed additional data as necessary (i.e., websites related to specific projects, videos of JOGL meetings, and documents created by JOGL members). In total, we analyzed approximately 1,300 pages of data. Results were analyzed using a codebook which was developed inductively by all four authors based on the principles of grounded theory (Belgrave and Seide 2019; Strauss and Glaser 1967), and iteratively revised through group discussion.

### Phase 2: interviews

To gain a deeper understanding of the data gathered in Phase 1, and to clarify and extend our primary observations, we conducted hour-long interviews with those involved with JOGL. We recruited from three subgroups of interest: those involved with formulating biosafety guidelines or those who participated in biosafety discussions (“biosafety group”), those conducting specific COVID-19-related projects (“project participants”), and those holding leadership positions at JOGL (“JOGL leadership”). Detailed information about sampling and recruitment is presented in Appendix 2. The interview guides (Appendix 3) were informed by the data gathered in Phase 1, as well as by preliminary consultations with those involved in either studying or conducting DIYbio. Specific interview questions varied by subgroup, and assessed participants’ prior involvement with DIYbio, their experiences with JOGL, their involvement in or awareness of biosafety guidelines, ethical issues they encountered, and attitudes toward ethics in DIY biology. All interviews were conducted by a single interviewer (AP) via webconference and audio-recorded with participant’s consent. Participants were offered a $40 Amazon e-gift card for completing the interview as compensation for their time.

A total of 31 interviews were conducted between February and June 2021 across the biosafety group (n = 13), project participants (n = 12), and JOGL leadership (n = 6). Most interviewees were male (58.1%; n = 18) and the majority had prior experience with DIYbio (80.6%; n = 25). Interviewees were located in North America (n = 20), Europe (n = 6), Africa (n = 2), Asia (n = 1), or reported splitting time between multiple locations (n = 2).

All interviews were transcribed via SpeechPad. The interview codebook was developed inductively following an examination of a subset of transcripts by two authors (AP, RC), and iteratively revised by all four authors. During the first round of coding, two coders (AP, RC) categorized all relevant text through inductive coding using the qualitative analysis software Dedoose; these excerpts were exported and cleaned in Excel. Next, two coders (AW, RC) used code mapping, the process of theming or recategorizing groups of codes, to analyze and combine first-level categories that were identified in the initial coding round. All themes that emerged were independently assessed for accuracy and consistency by two coders (AW, RC). Any disagreements were resolved through discussion and by referencing other data samples.

Both Phase 1 and Phase 2 of this study were deemed exempt from review by the University of Pennsylvania Institutional Review Board.

## Results

Five primary themes related to biosafety emerged from our data. First, biosafety on JOGL was approached in a unique way: namely, JOGL was the first global DIY biology initiative to have a Biosafety Advisory Board, as well as a detailed set of biosafety guidelines that applied across multiple groups and projects. Second, while there was recognition that these achievements were unprecedented in the realm of open science, those involved with the Biosafety Board or with drafting the guidelines did not view their efforts as particularly novel, but rather as the natural culmination of nearly a decade of work to enhance biosafety practices within DIY biology. Third, both on Slack discussions and in interviews, there was disagreement regarding whether the Board should have an advisory role or provide mandatory oversight of projects. Fourth, in at least one instance, JOGL practiced a kind of ethical gatekeeping, excluding a potential project because it was not in keeping with ethical limits defined by the Biosafety Board. Fifth, individuals differed in their perception of the utility of the biosafety guidelines: while there was an awareness of them among project participants, members of the Biosafety Board and JOGL leadership questioned whether they had provided real-world value to individual projects.

Each of these themes are discussed below, drawing from both ethnographic and interview data. Direct quotes are attributed to interviewees according to subject number and group (i.e., “B1” is a biosafety group member, “P1” is a project participant, and “J1” is a member of JOGL leadership). Given the international nature of our sample, some interviewees were not native English speakers. Attributions are not provided for Slack quotations to protect participants’ privacy.

### Theme 1. A first for DIY science: a global biosafety board and biosafety guidelines

The early days of the OpenCovid19 Initiative were full of promise: thousands of people interested in open science came together virtually to fill gaps in SARS-COV-2 diagnostics, prevention, and treatment. Whereas previous open science efforts had been disjointed and fractured, with individuals working locally on different projects, in March 2020, individuals focused their attention, jointly, on common problems related to COVID-19. For some in JOGL leadership, the sudden surge of interest and feeling of global unity was emotionally moving; one described it as a quasi-religious experience, as a “feeling of togetherness that you can touch somehow; it was amazing” (J2).

At the same time, however, those involved in the OpenCovid19 Initiative began to worry about the prudence and safety of some of the projects that were being proposed on JOGL. One interviewee noted that upon seeing the details of some of the diagnostic and vaccine projects, “my tentacles that this could go wrong were kind of tingling.” (B4). Another recalled:

I think really early on people were talking about like, "Let's go out and collect samples," I think of, like, swabs, swabbing other places, in essence, which would have brought, you know, the virus into their facilities. And really quickly, people started raising alarm bells about being like, "Hold on. Let's think that through" (B2).

Because JOGL was an open platform, it presented a specific problem: there was no clear vetting mechanism, which meant anyone could join and propose a project. One interviewee noted that while an open platform like JOGL welcomed everyone, there was a concomitant “risk of welcoming, not necessarily evil doers, but incompetent doers” (B9).

On March 11, 2020, just a few weeks after the launch of the OpenCovid19 Initiative, a weekly JOGL community Zoom meeting was held during which Thomas Landrain, the co-founder and CEO of JOGL, proposed the creation of a Biosafety Advisory Board that could both review projects and serve as a community resource to answer questions (JOGL 2020c). The idea was met with enthusiasm by others at the meeting, and that same day, a Slack channel devoted to biosafety was created by JOGL’s program coordinator, with its description stating that it is was dedicated to “discussions/questions about biosafety and biosecurity concerns for the program.”

On the channel, Landrain noted that he foresaw the Board as having three main goals: publishing general recommendations, reviewing projects within the initiative and providing guidance and warnings, and answering biosafety and biosecurity questions asked by JOGL members. On the weekly call, an individual who had long been involved in biosafety in DIY science was nominated to serve on the Board; she in turn recruited others, most of whom had been involved in previous biosafety endeavors in DIY biology. The Board included individuals such as Todd Kuiken, a researcher with extensive experience studying and advocating for enhanced biosafety practices within DIYbio; Chris Monaco, a microbiologist and lab equipment designer at the Center for Disease Control (CDC); and various founders and directors of community labs, among others.

Efforts coalesced quickly, and by early April, the ten-member Board had published a fifteen-page document outlining biosafety guidelines for the OpenCovid19 Initiative (JOGL 2020b). The guidelines provided both general information about biological risk assessment as well as specific recommendations (e.g., for the use of positive controls and sample collection). They also specified that no human or animal testing should be performed without prior approval, and that testing should not be offered or marketed to the general public. The guidelines were the first detailed, public biosafety guidance to have emerged from the DIY science movement.

The speed with which JOGL’s efforts toward biosafety impressed even the Board members:

I was happy to see how quickly the folks that sort of were in that JOGL community in the beginning, how quickly they recognized that they needed some additional guidance around biosafety, and how really fast they formulated that committee in case issues came up. I was really glad to see that happened as quickly as it did (B2).

While there had been previous efforts toward biosafety within DIY science, JOGL’s version was unique for several reasons. First, while prior endeavors toward guiding the practice of biosafety had largely taken place at community levels (Guerrini et al. 2019a, b), the international nature of JOGL meant that the Biosafety Advisory Board and guidelines spanned boundaries in ways that previous efforts had not. Second, local efforts toward biosafety often occurred informally: the head of a community lab might instruct a new member in best practices in their specific lab by training them, rather than referring to an established manual. The JOGL biosafety guidelines, however, represented a kind of formal, written knowledge of biosafety practices, albeit ones geared specifically toward COVID-19 efforts. Third, the Board and its guidelines had a measure of external credibility, as Dee Zimmerman, the former President of Association for Biosafety and Biosecurity (ABSA), the largest international biosafety association, was a Board member and is listed as a coauthor of the biosafety guidelines.

These accomplishments were recognized as significant by those involved in DIY biology. One Board member noted:

The fact that the OpenCovid19 Initiative formed a biosafety group composed of people from around the world in open science communities and then they actually made a deliverable happen is huge. Because that wasn't able to be done very well in the past (B6).

Another Board member, who has long been involved in biosafety efforts in DIY biology, described JOGL’s biosafety group as a fundamental advance in the sophistication with which open science efforts had evolved in the last decade because “the community itself [recognized]” (B2) the need for ethical oversight and subsequently developed an organizational architecture suited for that purpose.

### Theme 2. Natural culmination of a decade of efforts

Despite the uniqueness of JOGL’s achievements in the realm of biosafety, members of the Board did not see their efforts as extraordinary. Rather, as one put it, “it was just a culmination of years of people working on these things” (B2). Some drew a direct line between JOGL’s biosafety endeavors in 2020 and early efforts toward biosafety in open science, which included the development of the Codes of Ethics at European and U.S. DIYbio workshops in 2011 (Eggleson 2014; DIYBio.org 2011). Though these codes were relatively limited in scope and the relevant mention of biosafety comprised just four words (“Safety: adopt safe practices”), it was the first time that the DIY science community had publicly and formally recognized that biosafety should be a guiding principle.

The ensuing years saw other endeavors related to biosafety in open science, many of which were supported by external funding. In 2012, the Federal Bureau of Investigation (FBI) held a joint meeting with members of the DIY biology community, in which it paid for individuals to attend and discuss issues related to biosafety (Scroggins 2013; Lempinen 2011). The Wilson Center funded a study on DIY biology through its Synthetic Biology Project (Grushkin et al. 2013; Kuiken et al. 2018), as well as the “Ask a Biosafety Expert” feature on DIYbio.org in 2013–2014, an open forum where anyone could ask biosafety-related questions. A grant from Open Philanthropy allowed Todd Kuiken and Dan Grushkin, the co-founder of New York City’s community lab Genspace, to hold a three-day biosafety “boot camp” in 2019 for DIY Biology and community labs, which was presented in partnership with ABSA (Baltimore Under Ground Science Space 2019; ABSA 2019). This same funding also supported their endeavors to evaluate community biosafety procedures and develop best practices. In October 2020, approximately six months after JOGL’s guidelines were released, they and several additional co-authors released a 250-page Community Biology Biosafety Handbook at the Global Community Bio Summit, an annual global DIYbio meeting (Armendariz et al. 2020).

In reflecting upon JOGL’s efforts toward biosafety, members of the Board described the Board and its biosafety guidelines as coming together “pretty easily” (B1), “naturally” (B3), and “smoothly” (B4). Interviewees pointed to four main factors that contributed to the ease with which the Board and guidelines came to fruition. First, Board members and JOGL leadership had extensive experience with issues related to biosafety, both in institutional settings as well as in DIYbio. Board member Kuiken, for example, had conducted the Code of Conduct DIYbio workshops, and had led the Wilson Center reports and Open Philanthropy efforts; Board member Jorgenson was the co-founder of the first community DIY science lab in New York City; and Elena Perez-Nadales worked as a biomedical researcher at Maimonides Biomedical Research Institute in Cordoba, Spain. JOGL co-founder Landrain had led the DIY Bio Congress in Paris (Synenergene 2014). This gave them not only experiential expertise but also access to written biosafety documents. As one Board member noted:

And yeah, of course, from my experience here at the [redacted] with making [redacted], I have whole like SOPs, standard operating procedures of how to, you know, double glove and all this kind of stuff... Yeah, I used some of my old things (B3).

The second main factor was that Board members were individuals who, for the most part, knew each other and had established positive working relationships, largely due to their previous experience with biosafety in DIYbio. In interviews, they typically referred to each other by first name, and often mentioned the esteem and trust in which they held their fellow Board members. These prior working relationships were also what allowed them to coalesce quickly as a group, as they were able to quickly recruit others to the JOGL efforts. One Board member recalled reaching out to other co-authors of the Community Handbook after hearing about JOGL’s interest in biosafety guidelines:

As soon as I saw the announcements and had realized the type of work that some of the JOGL community were interested in doing, I emailed the other biosafety authors and said, "Hey, have you seen this? Maybe we should at least like, definitely make sure they have access to the chapters, sort of, even if they're not all online right now as public links. And, you know, would it be worth doing something?" And then I think we agreed on an email thread that that would be worth doing (B4).

The third factor cited as facilitating the rapid formation of the Board and the development of the guidelines was prior relationships with outside experts. Because Kuiken and others had already interacted with ABSA at the biosafety boot camp, the Board had a direct line to the organization, and to its past president, Dee Zimmerman. This enabled the Board to obtain informal advice and feedback, thereby ensuring the guidelines they had put together were valid and credible. As one Board member noted:

Connections that we had through ABSA… I think that was pretty huge having that. I don't know if I'd say it's an endorsement, but having a name like ABSA, be, I guess, somewhat affiliated with it, I think, gave it a lot of credibility… (B3).

The fourth factor was that in March 2020, when JOGL expressed interest in creating COVID-19 biosafety guidelines, there had already been a significant amount of work put into the drafting of the Community Biosafety Lab Handbook that would be released just months later:

Most of the work [on the handbook] had been completed at that time. I think the copy editing was still ongoing, and like it hadn't been put into one giant document at that moment… So, definitely, having the draft or un-copy-edited version of the biosafety handbook was helpful (B4).

Many Board members mentioned that they drew largely on this handbook when crafting the biosafety guidelines. While only four authors out of the dozen on the Community Biosafety handbook were also on JOGL’s Biosafety Advisory Board, drawing on the handbook effectively allowed the Board to benefit from the efforts of the larger Community Biosafety handbook group. However, Board members also sourced additional material from other outlets, as the handbook did not contain specific information related to COVID-19. On the Slack biosafety channel, Board members posted links to guidelines from the Center for Disease Control (CDC), the World Health Organization (WHO), and the National Institutes of Health (NIH); in interviews members confirmed drawing on this material as well as additional documents, such as those published by Public Health England.

Thus, while the Biosafety Advisory Board and guidelines were a significant achievement for open science, Board members made it clear that their work was indebted to previous efforts over the last decade, many of which had been externally funded. The accomplishments of JOGL in the realm of biosafety therefore built on prior relationships within the community and with outside experts, as well as both formal and informal bodies of knowledge related to biosafety.

### Theme 3. Role of the Biosafety Advisory Board: advisory vs. mandatory

When Landrain, the CEO of JOGL, had initially proposed the Biosafety Advisory Board, he saw it as having three main goals: publishing ethics guidelines, reviewing projects, and advising JOGL members. While those involved in biosafety were largely in agreement with the goals of establishing guidelines and advising JOGL members, the extent to which the Board should provide oversight proved to be a thornier issue. Some felt that the Board should act like a review committee, providing a seal or stamp of approval for JOGL projects. In this view, the Board would have the power to both approve projects and exclude them. As one member of JOGL leadership noted on the biosafety Slack channel:

Projects are free to join but if a project is following a dangerous road and its leaders ignore the warning of the board, I think it’s important to be able to say that they can’t be part of this initiative anymore.

Others, however, maintained that the Board should act in a purely advisory role. As the Board was forming in early March, one member summed up the debate on the biosafety Slack channel:

I think what needs to be decided on sooner rather than later is if this board is acting in a similar capacity to that of a review committee at an institution. Will projects need to come to us for approval before they proceed? Or are we simply providing guidelines, recommendations and access to experts that can answer questions and weigh in.

While on its surface, the question of the role of the Board was an administrative one, it also cut to the heart of a deeper issue: what modes of gatekeeping, if any, should be acceptable on an open science platform? Banning projects from participating in the OpenCovid19 Initiative would go against the radical openness that is critical to the ideology of DIY biology, yet including a potentially dangerous project could harm the reputation of JOGL and DIY science as a whole. One Board member described it as a delicate balancing act:

You’re trying to, balance that line, between, making sure that people are doing things safe, but also not preventing them from pursuing their ideas and interests (B1).

Most Board members ultimately favored an advisory role: one where the Board would provide guidance but not mandatory oversight. They maintained different justifications for holding this position. Some felt that there would not be “buy-in” from the open science community for mandatory oversight, and it would therefore not only be impractical but would also drive people away from JOGL:

From my standpoint, it's kind of, why alienate people when you... get the same sort of benefit from an advisory board that's giving that advice without saying, “You must do X, Y, and Z to do this” (B2).

Along similar lines, other Board members expressed hesitancy about mandatory oversight, either because they felt the Board lacked a legitimate claim to authority, or because they felt such authority would not have been widely accepted by members of the open science community. Still others felt that there was no clear mechanism or process by which the Board could have exercised its authority, as establishing a review system would have required a system of rules: regarding how projects would obtain initial approval from the Board, processes for projects that lacked approval or those that subsequently violated biosafety standards, and means of appealing Board decisions. Without a comprehensive system that outlined consequences for actions—and an elucidation of how such consequences would be enforced—some Board members felt that a mandatory review process would be unsustainable.

Others felt that the fundamental goal of biosafety guidance was not to be punitive, but rather to facilitate the conduct of research in as low-risk manner as possible:

It has to be supportive. And I can honestly say, in my career, there have been very, very few times where I have looked across at a researcher and said, "No. You cannot do this." I'll probably say maybe four or five times in my career because there's always a way that you can do it. The whole point of biosafety isn't to stop the research, it's to ensure that it's done safely (B7).

A final concern was related to accountability. If the Board approved a project, and that project ultimately caused some kind of physical harm, to what extent would Board members be liable? This was not only an issue for Board members from within the DIY biology community, but also one that could hamper the Board’s ability to recruit outside experts. As one Board member put it:

I was keen that the board was advisory I think, partly because we actually wanted to bring in some biosafety professionals who have actually got some experience in the field. And I personally felt like that, having something more formalized would be challenging to persuade them to do because there's quite a bit of exposure given the particular scenario... I mean, as soon as you assume some kind of formal responsibility, there is a kind of liability that you're accepting as well, to some extent (B4).

Ultimately, it was agreed that the Board would assume an advisory rather than an oversight role, due to the fact that most Board members held this position. As one member of JOGL leadership reflected:

They [the Board] came on knowing that it was mostly for advising and creating biosafety and biosecurity guidelines. And so, they didn't accept that position knowing that they could actually be accountable. So, it was clear that then this would become more of an advice that we at JOGL would actually use and execute. So, we would be the ones accountable for making decisions, as we are accountable when we are kicking out members out of the platform because that person is not behaving well (J5).

Thus, as will be discussed below, the responsibility for drawing lines with regard to biosafety—between who could participate and who could not—ultimately fell on JOGL, rather than on the Biosafety Board.

### Theme 4. Ethical gatekeeping

In positioning itself as a platform for those interested in open science, JOGL drew its membership largely from those who had previously participated in the DIY biology community. Indeed, on Slack, weekly JOGL calls, and throughout our interviews, individuals continuously referred to themselves and their peers as a “community.” But there were no criteria that conferred membership in the community, nor were there grounds for exclusion. As one interviewee put it:

I mean, the community is not a community. The community is a bunch of people who self-identify as being in the community. So, there's no way to keep people out of the community. But if people start to do things that are deceptive, dangerous, and under the name of being groundbreaking, but not really paying attention to science......it just becomes problematic for the entire community because the press loves a story about people acting dangerously (B5).

Because JOGL provided a platform for the DIY biology community, it was faced with a dilemma: should the organization itself draw boundaries to exclude potentially dangerous projects, or should it leave such decisions up to the community writ large? Some in JOGL leadership felt that it was not JOGL’s place to police unsafe projects—and that the community would naturally exclude those who were not interested in upholding the ethical norms of DIY biology. As one member of JOGL leadership put it:

The community that makes up JOGL is less likely to do or practice unethical experiments. And so even if there was one unethical person who had some idea, or maybe they didn't realize it was unethical… they may be able to connect with other people who do have expertise in what they're doing and realize that JOGL was not the platform for it because of the general culture there. And then they go off and do their own thing as a secret little group (B11).

Others in JOGL leadership, however, felt that informal social controls would not be sufficient to maintain order, and that JOGL, as an organization, needed to take on the responsibility of creating its own boundaries:

I think it was an argument between us [in JOGL leadership], like, "We need a code of conduct," and people are like, "Oh, no, don't worry, it will be okay." "Well, we need a code of conduct." I think, very practically, we needed one to be allowed as an organization to exclude people. The inclusion criteria of our community, there is none. But I think a code of conduct is a way of saying, "This is our principles, if you don't abide by it, then you leave." (J1)

Ultimately JOGL did create a code of conduct—outlining inappropriate behavior (harassment, violence, trolling) that were grounds for exclusion from the platform—and all new members of JOGL were required to check a box, agreeing to abide by it. However, the code did not encompass unsafe experiments, and since the biosafety guidelines were merely advisory, a violation of them was not grounds for exclusion.

JOGL’s policies were tested several months into the pandemic, when an open-source vaccine collectively known as RaDVaC began to discuss the possibility of joining the JOGL platform. Because RaDVaC had published the instructions for developing and self-administering a DIY COVID-19 vaccine on their website (RaDVaC 2022), some in JOGL worried that they were not upholding the ethical and safety norms of the community. As one Board member recalled:

To me, the most worrisome thing was when the RaDVaC people dunked in, because I felt that they aren’t actually following any of those guidelines that the DIY community follows, and that they were more interested in just getting more people to test their vaccine out on (B2).

By the time RaDVaC expressed interest in joining JOGL in late 2020, there had been no activity on the public Slack biosafety channel for months, and the Board was no longer in active communication about JOGL-related biosafety issues. There was therefore no formal assessment of whether RaDVaC’s approach to ethics and safety was in keeping with JOGL’s standards. However, a Slack channel was created dedicated to open-source vaccines, where one member of JOGL leadership expressed optimism about an open-source vaccine, but suggested a further conversation about what JOGL “could do to support your initiative, [and] how we could improve safety measures.” Soon after, an informal Zoom meeting was held among RaDVaC, JOGL leadership, and two members of the Biosafety Board who had become aware of RaDVaC’s desire to join JOGL. Regarding what transpired on the call, one interviewee recalled that JOGL “did actually not go with them except we didn't kick them out” (J5); another, however, recalled that JOGL “shut down” RaDVaC (B13). Following the call, members of RaDVaC were no longer active on JOGL’s Slack, and it was clear that JOGL and RaDVaC had not embraced one another.

Less clear, however, is what role the biosafety guidelines themselves played in denying RaDVaC a platform on JOGL. While the guidelines stated that no animal or human testing should be performed unless “you have gone through relevant approval processes,” there had been no official biosafety review. One interviewee who was on the call made no mention of the guidelines themselves as having been a reason for RaDVaC’s exclusion. But two others felt that having set out ethical norms—via the Biosafety Board having developed the biosafety guidelines—was key to being able to deny RaDVaC a prominent platform at JOGL. As they put it:

The RaDVaC projects which was, you know, very cool, you know, on paper because, you know, having...developing open-source vaccines is...would be really, really important...but the way they approached the problem was not compatible with the ethical limits that we had defined where there was no self-testing (J5).

JOGL kind of said no to them [RaDVaC] when they came up with that. And so, to me, that sort of shows the success of these programs [the Biosafety Board and guidelines] because when they're presented with sort of a project or an opportunity that clearly would go against those norms, they stuck to those norms and said, "No, thanks" (B2).

Thus, even after the height of the pandemic, JOGL practiced a kind of ethical gatekeeping. While there was no formal biosafety review, the process of defining ethical guidelines resulted in a decision to exclude a project that was not adhering to defined ethical boundaries.

### Theme 5. Utility of the biosafety guidelines

While the guidelines appear to have played a role in allowing JOGL to exclude projects, the extent to which they provided positive value to individual projects is less clear. Some Biosafety Board members and JOGL leadership doubted whether the community even was aware of them, noting they themselves couldn’t “even really find the guidelines” (B13) and wondered how anyone else would find them. In addition, both Board members and JOGL leadership repeatedly highlighted the lack of attention to dissemination of and follow-through regarding the guidelines. Some felt that there should have been additional efforts to increase awareness of them. As one Board member stated:

I don't think there's been any sort of accountability if that makes sense…. Or any sort of, like, follow up to make sure that people are following them. I think it's important that they [biosafety guidelines] are there and that people have access to them but I don't know if... Maybe there could be a little bit more, like, push to let people know that they're there (B1).

In a similar vein, some in JOGL leadership felt that the Board had ended its efforts too early, and that it was the Board’s responsibility to have been more engaged throughout the lifetime of JOGL:

The fact that I know they [the Board] aren't active anymore, haven't been for a long time, to me says that maybe they felt once the guidelines were produced, that their job was done. But the job wasn't done because then they had to get the information out and visible. (J6).

One member of the Board acknowledged the validity of these concerns, but felt that the underlying problem was related to the lack of time and funding, not necessarily the personal dedication of Board members:

Just having a link to a manual is not enough. Having an advisory board there helps, but is that enough? Is it really [enough] knowing if people are trained? No. But do we have the resources to do all that? No. (B2).

Although most Board members and JOGL leadership believed that the JOGL community was likely unaware of the biosafety guidelines, this view was not supported by our interviews with project participants: of the 12 individuals interviewed, nine reported being aware of the guidelines. This awareness may have been due to one significant step JOGL had taken to make individuals aware of the biosafety guidelines. The organization had raised money from the AXA Research Fund (AXA 2020), and in April 2020, it began to offer “micro-grants” of several thousand dollars for individual projects. As part of the grant application process, JOGL required applicants to check a box, acknowledging that they had read the biosafety guidelines and agreed to adhere to them (JOGL 2021).

This simple endeavor—requiring any individual applying for a JOGL microgrant to pledge to abide by the biosafety guidelines—appears to have had a significant effect on awareness. Eight of the nine project participants who reported being aware of the guidelines had also been funded by a JOGL microgrant, whereas all three who were not aware of the biosafety guidelines had not received any JOGL grant.

It is worth noting, however, that awareness itself is not a measure of utility or value. None of the project participants mentioned using the guidelines. For some of the projects—such as those that used machine learning to develop software applications, or those that focused on educational projects—the biosafety guidelines themselves were irrelevant to their project, as their projects did not involve the use of biological samples.

The more significant explanation for the questionable utility of the guidelines, however, may have rested on the fact that most projects—running on the good will of volunteers and lacking any resources—never progressed to any kind of advanced stage where biosafety concerns would have been relevant. And of the few projects that had advanced, several interviewees noted that such projects were led by individuals who had significant prior biology experience or an “institutional foot” in the door (J2). Thus, they felt that those whose projects had reached an advanced stage were already practicing good biosafety practices—that “they're doing what it says in those guidelines, whether they know it or not” (B2). As one Board Member put it:

Most of the people who would need them [the guidelines] sort of knew it already… I think probably everybody who needed them, knew (B3).

Thus, while the project leaders’ awareness of the biosafety guidelines was likely influenced by the requirement to acknowledge them in JOGL’s microgrant application, there was agreement among Board members and JOGL leadership that more could have been done to disseminate them. The guidelines did not appear to play a role in educating newcomers about biosafety practices, but rather, as discussed in the previous section, played a more symbolic role in upholding the ethical norms of JOGL.

## Discussion

While prior scholarship has highlighted ethical issues in DIY science and assessed participants’ attitudes toward ethics (Guerrini, Trejo, et al. 2020; Trejo, et al. 2021; Guerrini et al. 2021; Rasmussen 2021, 2017), the present study is, to our knowledge, the first to assess how DIY biologists navigated one ethical issue, biosafety, in real-world practice. Our results align with previous findings showing that rather than proceeding recklessly and with disregard for risks, most in the DIY biology community value safety and endeavor to prioritize it in their practices (Grushkin et al. 2013; Kuiken et al. 2018; Meyer and Vergnaud 2020; Lim 2021; Trejo et al. 2021; Seyfried et al. 2014). Specifically, in this study, we demonstrate how leaders of the JOGL platform, the main online hub for DIY science during the COVID-19 pandemic, quickly recognized the potential biosafety risks and actively tried to build the infrastructure to help facilitate the safe conduct of research. The fact that an international organization like JOGL succeeded in forming a Biosafety Advisory Board and publishing a set of biosafety guidelines—ones that had a measure of external credibility due to the participation of outside experts—marks a significant achievement in the realm of DIY science.

These accomplishments, however, did not come in a void; they built upon a decade of prior efforts related to open science and biosafety that were largely supported by external funding. These past endeavors had resulted in both outside partnerships and resources that facilitated the rapid development of JOGL’s biosafety guidelines. That the DIY Biology community not only welcomed relationships with experts, but also actively sought them out, is in line with the findings from Trejo et al. (2021) that the community values such partnerships, as long as outsiders respect the independent nature and culture of DIYbio. In that sense, our findings lend empirical support to policy proposals that have called for transparent and collaborative engagement on the part of experts and government agencies to enhance the ethical conduct and safety of DIY science (Guerrini, Sherkow, et al. 2020).

Still, compared to the manner in which biosafety is regulated in institutional settings, JOGL’s Biosafety Board lacked teeth: by maintaining a purely advisory role, it lacked the authority to provide any kind of significant oversight. The Board was also not active for long, likely due to both the waning of interest of JOGL members in COVID-related projects by late 2020 (as mainstream science was successfully developing diagnostic tests and vaccines) as well as the lack of progression of projects to stages where biosafety concerns would have become relevant. The reluctance of the Board to adopt a mandatory approach to ethical oversight is not surprising, as a prior study assessing DIY biologists’ preference for ethical oversight found that participants were “especially opposed to mandatory, rule-based oversight mechanisms” (Trejo et al. 2021). Many Biosafety Board members in our interviews appeared in-tune with this sentiment, as their reluctance to adopt an authoritative approach was driven by their perception that it would not have been accepted by the wider DIY biology community.

Even though the power of JOGL Biosafety Board’s was limited, its output—the biosafety guidelines—appears to have provided a formal foundation for JOGL leadership to exclude an open-source vaccine project. While there was no assessment by the Biosafety Board of the open-source vaccine project—and it is possible that even without the biosafety guidelines, JOGL would have excluded the project—the guidelines offered JOGL leadership a concrete expression of ethical norms and standards, ones that were developed by representative members of the community and outside experts. The guidelines themselves therefore had a measure of authority, and could be referenced in any exclusion decisions.

That JOGL was able to exercise ethical gatekeeping was likely due to two structural features. First, it provided access to an international network of DIY science projects and peers through an online platform that it controlled. Second, it also acted as a funder, providing modest microgrants for COVID-19 projects. At both of these points—access to the platform and the provision of funding—JOGL required individuals to certify that their behavior and projects adhered to certain ethical norms and standards. In this way, JOGL could define borders between who could participate and who could not. In at least one respect, JOGL attempted to have those lines drawn by representative members of the community (i.e., through the development of the biosafety guidelines). In the future, recognition and identification of sites of ethical gatekeeping may be helpful in terms of enhancing the ethical conduct of DIY science.

The fact that there was ethical gatekeeping on JOGL does not mean that actors not allowed to be part of the “community” will cease their efforts, but rather such efforts may occur in more isolated settings, without the full support of (or enculturation from) those involved DIY biology. Thus, efforts to support biosafety within DIY biology should focus not only on improving practices within the community, but also identifying and addressing risks from splinter groups or individual actors.

This project has a number of limitations. First, because we had access only to public Slack channels, it is possible that additional conversations regarding ethics and biosafety occurred privately (i.e., via direct message or email) and were therefore not captured by our methods. Second, since most projects did not reach the stage of encountering significant biosafety concerns, our data on how individual project members may have resolved biosafety issues are limited. Third, as our interviews were conducted nearly a year after the height of activity on JOGL, participants’ recollections may have been incomplete; however, whenever possible, we confirmed participants’ recollections with data from our digital ethnography. Fourth, our study focuses on a subset of individuals involved with DIY biology and may therefore not be generalizable to the wider DIY biology community.

## Conclusion

The present study reinforces the findings of previous scholarship that has pointed to a strong interest among DIY biologists in incorporating safe and ethical practices—albeit in a way that is respectful of their core values of openness and flexibility. For the DIY biology community, our findings point to the importance of community-developed standards and attention to sites of ethical gatekeeping; for those outside it, such as policymakers, our study points to the importance of fostering partnerships between outside experts and DIY biologists. Our results also point to the critical role that external funding has played in providing the community with resources to develop approaches and training for addressing biosafety issues. Finally, given the challenges of regulating non-establishment research, and the public health interest in addressing any concomitant potential biosafety risks, it is imperative that future research continues to examine the practices of DIY science.

## Supplementary Information

Below is the link to the electronic supplementary material.

Supplementary file1 (DOCX 109 kb)
