# Supplementary material for: Navigating biosafety concerns within COVID-19 do-it-yourself (DIY) science: an ethnographic and interview study
Source: Biosocieties. 2023 Mar 28:1–22. Online ahead of print. doi: 10.1057/s41292-023-00301-2 (PMC10042665; doi:10.1057/s41292-023-00301-2)
Supplement: Supplementary file 1 — Supplementary file1 (DOCX 109 kb) [file 41292_2023_301_MOESM1_ESM.docx]

**Appendix 1:**

**Phase I Digital Ethnography Keywords**

To conduct a digital ethnography of JOGL’s Slack channel, we iteratively developed a list of biosafety-related keywords, as well as broad-brush terms related to general ethics. This process was informed by pilot research that involved informal conversations with members of the DIY Biology community, through which we identified biosafety-related topics on the JOGL Slack channel (e.g., handling live samples of the SARS-CoV-2 or the use of personal protective equipment within labs.) An initial set of keywords was generated by the four authors, iteratively revised through group discussion, and tested repeatedly for relevance, until all authors concurred with the final list. In total, we identified 23 keywords that resulted in the most relevant biosafety discussions on Slack (Table S1).

Next, deidentified transcripts of all COVID-19 Slack communications between March 1, 2020 and August 31, 2020 were provided to us by JOGL. We retrospectively searched all transcripts for posts that contained any use of the keywords. All posts were reviewed by two authors to ensure relevancy; posts were excluded if they were not written in English. For all posts deemed relevant, we captured/extracted the initial post, any subsequent threads, as well as the conversational context. These excerpts were reviewed by two authors, and duplicates were excluded. A search of all keywords resulted in 1,969 total hits and 1,131 pages of data.

**Table S1. List of keywords utilized for JOGL Slack searches.**

| **Central ethical issues** | **Keywords** | **# of results** | **# of pages of data generated** |
| --- | --- | --- | --- |
| Biosafety | Biosafety | 132 | 38 |
|  | Safety | 78 | 27 |
|  | Vaccine | 107 | 36 |
|  | Treatment | 192 | 136 |
|  | Approval | 182 | 219 |
|  | Harm | 18 | 16 |
|  | Warn* | 39 | 13 |
|  | Safe* | 111 | 82 |
|  | Trust | 40 | 17 |
|  | Danger* | 27 | 35 |
|  | Risk* | 135 | 100 |
|  | Hurt* | 14 | 5 |
|  | Infectious* | 81 | 53 |
|  | Contaminant* | 51 | 51 |
|  | Exposure* | 27 | 13 |
|  | Infect* | 268 | 205 |
| General ethics | Advisory | 26 | 10 |
|  | Ethic* | 84 | 47 |
|  | Principle | 45 | 29 |
|  | Guidance | 55 | 24 |
|  | Advisory board | 18 | 14 |
|  | Open science | 185 | 114 |
|  | Transparency | 54 | 27 |
| **TOTAL** | | **1969** | **1311** |

* Indicates that all variations of the search term were included in the final search results. E.g., “infect*” also includes “infection”, “infectious”, “infecting”, and “infected”.

**Appendix 2:**

**Phase II Detailed Interview Methods**

Based on the data gathered in Phase 1 of the study, we recruited from three subgroups of interest: those involved with formulating biosafety guidelines or those who participated in biosafety discussions (“biosafety group”), those conducting specific COVID-19 related projects (“project participants”), and those holding leadership positions at JOGL (“JOGL leadership”). Eligible participants were either sent a brief direct message (DM) on Slack or a recruitment message using the email address associated with their Slack account. Two additional outreach attempts, separated in time by at least one week, were made to contact participants who did not respond to recruitment requests. Sampling methods for each subgroup are described below.

Biosafety Group: A core biosafety group was identified using the biosafety Slack channel. This channel provided a dedicated space for JOGL members to have discussions and ask questions about biosafety and biosecurity concerns. Recruitment requests were sent to all members of the Biosafety Advisory Board (n=10) as well as general users who posted messages of substance (i.e., not spam or general JOGL announcements) in the biosafety Slack channel (n=7).

The total response rate for the biosafety group was 13/17 (76.4%); interviewees were comprised of both Biosafety Board Members (n=7) and non-members involved in biosafety-related conversations on Slack (n=6).

Project Participants: All n=107 projects listed under the OpenCovid19 Initiative on the JOGL website (https://app.jogl.io/program/opencovid19?tab=projects) were screened by two authors (AP and RC) in March 2021. Duplicate projects (n=15) and those published in a language other than English (n=4) were excluded. Given that we aimed to assess projects that encountered ethical issues—and projects that had little or no activity likely did not encounter any ethical issues—we excluded projects that appeared to have little or no activity on their respective Slack channels (n=9), did not have a Slack channel (n=21), and projects with only one member (n=25).

For the remaining 33 projects, we extracted information from their JOGL pages about their project’s leaders and contact information. Two individuals were the sole project leads listed for five projects; these individuals were therefore contacted only once. Another two project leaders had already been interviewed in the biosafety group, and as a result were not contacted again. In total, we sent recruitment messages to project leaders from 27 unique projects. For the projects that listed more than one creator (n=8), a random number generator was used to select which project leader to recruit. In the event that there was no response from the selected participant, a second participant was selected using the random number generator (n=6). In total, we invited 33 individuals across 27 projects. The total response rate for the project participant group was 12/33 (36.3%).

JOGL Leadership: Eighteen individuals were listed on the JOGL leadership website (https://jogl.io/) as of April 2021, in roles such as community engagement intern, lead frontend developer, junior UX/UI designer, and peer review coordinator. We excluded individuals who worked in back-end roles related to the JOGL platform (such as software development and product management; n=4), or those who had joined the organization after our data collection period ending in August 2021 and thus likely had limited experience with the OpenCovid19 Initiative (n=6). Recruitment emails were sent to the remaining n=8 members of the core leadership team who were involved in the day-to-day operations of the platform, such as the co-founders, communication managers, and logistical coordinators. The total response rate for JOGL leadership was 6/8 (75.0%).

**Appendix 3:**

**Biosafety Group Interview Guide**

1. **JOGL Introduction and Involvement**
   1. How did you first hear about JOGL?
   2. Why did you get involved with JOGL?
   3. Which projects have you contributed to, if any?
   4. Overall, how has your experience been with JOGL?
2. **Background**
   1. Before JOGL, what was your prior experience, if any, with DIY Bio?
   2. What is your training and background?
   3. What is your current position?
   4. What is your prior experience, if any, with biosafety considerations?
3. **Formation of the Biosafety Advisory Board**
   1. How did you become involved?
   2. Do you think there was a need for a biosafety advisory board? Why?
   3. How much effort have you devoted to the Biosafety board?
   4. What do you understand to be the goals of the Board?
   5. What were some of the areas of *agreement* amongst board members in terms of goals, if any?
   6. What were some of the areas of *disagreement* amongst board members in terms of goals, if any?
   7. On Slack there seemed to be discussion about whether the board would just give advice or issue formal approvals for projects. What were your views about this issue? Why?
   8. What types of biosafety questions did you expect from the JOGL community, if any?
4. **Developing the Biosafety Guidelines**
   1. How involved were you in the development of the JOGL biosafety guidelines?
5. Were there other documents or standards that you looked to model these guidelines on?
6. In your opinion, what factors helped in the development of the guidelines document?
7. In your opinion, what factors may have impeded the development of the guidelines document?
8. Is there anything you think is missing from the guidelines?
9. **Perceived Success of the Board**
   1. Do you think that the JOGL community was aware of these guidelines?
      1. If yes → Why do you think that is? Can you provide an example?
      2. If no →  Why do you think that was the case?
         1. What do you think could have helped the biosafety guidelines reach the community?
   2. Do you think the guidelines have been helpful to the JOGL community?
      1. If yes → why do you think that is? Can you provide an example?
      2. If no → Why do you think that was the case? What do you think could have made the biosafety guidelines more helpful to the community?
   3. Have people approached the board with biosafety concerns?
      1. If yes → Can you elaborate? Are these the types of concerns you expected?
      2. If no → It sounds like not that many people approached the board with ethical concerns or questions. Why do you think that is?
   4. What would you have done if you’d heard through the grapevine of a biosafety issue that wasn't brought to the board?
   5. Previously you mentioned [] and [] as goals of the biosafety board. Do you think the board has accomplished these goals?
      1. Why or why not?
   6. Do you feel that there were additional goals that the biosafety board should have taken on? If so, can you elaborate?
   7. Do you feel that the Biosafety Board has been a successful endeavor?
10. **Looking Ahead**
    1. What advice would you give someone setting up a body like a biosafety board in open science initiatives in the future?
    2. Is there anything that would’ve made it easier to get the biosafety board up and running?
    3. Is there anything that could have been improved for the biosafety board to have been more effective?
    4. What more do you think a future biosafety board should do?
11. **General Ethics/JOGL**
    1. What ethical issues, if any, have arisen over the course of your involvement with JOGL?
    2. Looking ahead, do you anticipate ethical concerns arising with regard to any projects on JOGL?
    3. In terms of open science as a whole, are there areas where you feel that it could benefit from additional ethical guidance?
    4. The interview is now complete. Do you have anything else you would like to add

**Project Participant Interview Guide**

1. **JOGL Introduction and Involvement**
   1. How did you first hear about JOGL?
   2. Why did you get involved with JOGL?
   3. Which projects have you contributed to, if any?
   4. Overall, how has your experience been with JOGL?
2. **Background**
   1. Before JOGL, what was your prior experience, if any, with DIY Bio?
   2. What is your training and background?
   3. What is your current position?
3. **Project-related questions**
   1. Tell me about the project(s) you were working on for JOGL.
   2. What was the goal of the project?
   3. How much effort have you devoted to the project?
   4. Were you able to participate as much as you wanted to in your project?

If no → Why not?

- 1. Tell me a bit about where the project is now.
  2. In your opinion, what factors helped facilitate the progress of your project?
  3. In your opinion, what factors may have impeded the progress of your project?
  4. Do you feel that your project has been a successful endeavor?

1. **Biosafety Questions**
   1. Does the project that you were working on present any biosafety concerns?
   2. Were you aware that there was a JOGL biosafety board and Slack channel?
   3. Were you aware of the JOGL biosafety guidelines?
      1. If yes → How did you hear about them?
      2. If yes → Did you need to consult them during your project?
      3. If yes → What do you think could have helped the biosafety guidelines reach the community?
   4. Are you aware of other DIY Bio biosafety efforts? Have you participated in any of them?
   5. Do you think there is a need for additional biosafety resources or guidance for DIY Bio?
2. **Ethics Questions**
   1. Did your project run into any ethical issues?
      1. If yes → What ethical issues did you encounter?
   2. Outside of your project, did you hear about any other projects that ran into ethical issues?
      1. If yes → What ethical issues did they encounter?
   3. Looking ahead, do you anticipate ethical concerns arising with regard to any projects on JOGL?
   4. In terms of open science as a whole, are there areas where you feel that it could benefit from additional ethical guidance?
3. **Peer Review**
   1. Did you participate in the peer review process?
   2. Did you feel like it worked the way it was supposed to?
   3. What, if anything, would you change about it?
   4. Have you seen a peer review process anywhere else in the DIY community?
      1. If yes → Can you elaborate on what those looked like?
4. **Looking ahead**
   1. Did you get what you hoped to get from JOGL? Why or why not?
   2. What advice would you give someone setting up a project like this in future?
   3. What do you think has been unique about JOGL as compared to other DIY Bio and open science initiatives?
   4. What obstacles stand in the way of greater contributions to science from DIY communities?
   5. The interview is now complete. Do you have anything else you would like to add?

**JOGL Leadership Interview Guide**

*Questions varied depending on whether the interviewee was one of JOGL’s co-founders or a non-co-founder member of JOGL leadership.*

1. **JOGL Introduction and Involvement**

*→ [For non-co-founders]*

- - - - 1. How did you first hear about JOGL?
        2. Why did you get involved with JOGL?

*→ [For co-founders]:*

- - - - 1. Why did you decide to create JOGL?
        2. Did you see a specific need, or gap, that JOGL would fill?
        3. What has your role been at JOGL?
        4. Which projects have you contributed to, if any?
        5. Overall, how has your experience been with JOGL?

1. **Background**
   1. What is your prior experience, if any, with DIY Bio?
   2. What is your training and background?
   3. What is your current position?
2. **JOGL Organizational Structure and Governance**

*→ [For co-founders]:*

- 1. Can you tell me about the organizational structure of JOGL?
  2. Why did you set it up this way?
  3. Do you think this has been working?
  4. Is there anything that could have been improved, in terms of the organizational structure of JOGL?

*→ [For non-co-founders*]:

- - - - 1. JOGL was set up with a specific organizational structure, in that it’s not totally horizontal, but there is some hierarchy. Do you think this structure has worked?
        2. Do you think there’s anything that could have been improved?
        3. Have you been at all concerned about personal liability or responsibility, given your role at JOGL?

1. **Biosafety Questions**
   1. What is your prior experience, if any, with biosafety considerations?
   2. Do you think there was a need for a biosafety advisory board? Why?
   3. On Slack there seemed to be discussion about whether the board would just give advice or issue formal approvals for projects. What were your views about this issue? Why?
   4. What types of biosafety questions did you expect from the JOGL community, if any?
   5. Do you think that the JOGL community was aware of the biosafety guidelines?
      1. If yes → Why do you think that is? Can you provide an example?
      2. If no →  Why do you think that was the case?
   6. What do you think could have helped the biosafety guidelines reach the community?
2. Do you think the guidelines have been helpful to the JOGL community?
   - 1. If yes → why do you think that is? Can you provide an example?
     2. If no → Why do you think that was the case? What do you think could have made the biosafety guidelines more helpful to the community?
3. Do you feel that there were additional goals that the biosafety board should have taken on? If so, can you elaborate?
4. Do you feel that the JOGL Biosafety Board has been a successful endeavor?
5. What would you have done if you’d heard about a biosafety issue occurring on a JOGL project?
6. What more do you think a future biosafety board should do?
7. **Infrastructure Questions**
   1. What do you think has been unique about JOGL as compared to other DIY Bio and open science initiatives?
   2. JOGL set up a novel peer review process for its micro-grants. What were the goals of the peer review process?
   3. Do you feel like it worked? Why or why not?
   4. What, if anything, would you change about it?
   5. JOGL set up a code of conduct. What were the aims of setting up that code?
   6. Do you feel like it worked? Why or why not?
   7. What, if anything, would you change about it?
   8. In addition to peer review and code of conduct, what other systems or processes did JOGL put in place to help projects achieve their goals?
   9. What other systems/structures would you like to see put into place to help projects better achieve their goals?
8. **General Ethics/JOGL**
   1. Can you tell me about the RaDVaC situation?
   2. Have there been projects that have not been allowed onto the JOGL platform? If so, please describe.
   3. What ethical issues, if any, have arisen over the course of your involvement with JOGL?
   4. Looking ahead, do you anticipate ethical concerns arising with regard to any projects on JOGL?
   5. In terms of open science as a whole, are there areas where you feel that it could benefit from additional ethical guidance?
      1. If yes → How do you think JOGL might develop something like this?
9. **Looking Ahead**
   1. What are you most proud of about JOGL?
   2. Is there anything you wish would have been accomplished that wasn’t?
      1. If yes → What prevented that from being accomplished?
10. Can you think of any projects that were successful at making their way out into the world?
11. What obstacles stand in the way of greater contributions to science from DIY communities?
12. What advice would you give someone setting up open science initiatives in the future?
13. The interview is now complete. Do you have anything else you would like to add?
